# The global burden of perinatal depression: A call to action

**DOI:** 10.1371/journal.pmen.0000034

**Published:** 2024-06-04

**Authors:** Lakshmi Gopalakrishnan

**Affiliations:** Institute for Global Health Sciences, University of California San Francisco, San Francisco, California, United States of America; PLOS: Public Library of Science, UNITED KINGDOM

Pregnancy and childbirth are celebrated as milestones in a woman’s life, yet there exists a prevalent condition that is less frequently acknowledged and often misunderstood––perinatal depression (PND). This often-silent struggle, hidden from society’s and health system’s gaze, remains a formidable challenge for countless women despite effective interventions. Here, I shed light on the stark global disparities in the prevalence and management of perinatal depression, delving into determinants, challenges, and recommendations for the way forward.

## Focus needs to shift away from mere physical to include mental health

The focus of maternal and newborn health has traditionally been centered on physical well-being, primarily aimed at reducing newborn and infant mortality rates [1]. Maternal mortality had not gained recognition as a significant public health concern until the latter part of the 20th century [2]. While there were occasional mentions in international discussions, it wasn’t until 1985, spurred by a thought-provoking article titled "Where is the ’M’ in MCH?"[3] (referring to maternal and child health), that the international community began to seriously address the health of pregnant women. However, it’s imperative that our attention now also includes **mental health in MCH**. Managing perinatal depression is not only crucial for safeguarding maternal health, but untreated perinatal depression also has far-reaching negative consequences for families. It poses risks to the mother-infant bonding process, and can affect everyday care as well as a child’s cognitive, psychosocial, and motor development [4].

## The prevalence of perinatal depression is much greater in low-resource settings

Globally, perinatal depression affects one in four women [5] with the greatest burden in lower-and-middle-income countries compared to high-income countries [6], largely due to the limited accessibility to mental health professionals and resources [7], as well as factors such as social stigma [8] and reduced societal awareness. Prevalence of perinatal depression in low-and-middle-income countries (LMICs) is 30% antenatally [9] and 20% postnatally [10] with depression accounting for the greatest proportion of the burden associated with mental disorders in women of reproductive age [11,12]. Further, one in ten women [13] experiences suicidal ideation and faces an increased risk of suicide in the postpartum period, underscoring the significance of maternal mental health during the perinatal period.

Inequalities in health system factors from screening to treatment can elevate the risk of perinatal depression. In some settings, government programs on maternal mental health are virtually non-existent with extremely limited provision of mental health screening during pregnancy and postpartum periods [14]. Further, only one in 10 women diagnosed with perinatal depression receive treatment in LMICs, highlighting significant gaps in screening, access, and treatment [14]. Stigma around perinatal mental health conditions also remains a key barrier for perinatal women from seeking care [15].

## Various factors can be linked to perinatal depression

The development of perinatal depression is influenced by a complex interplay of determinants, which include psychological, biological, and social factors. These may include a history of depression or mood disorder, unwanted pregnancy, adolescent pregnancy, gestational diabetes, and other chronic medical conditions. [9,16] For instance, a systematic review including 37 studies and data from over 4,000 women found that 20% women with pre-existing bipolar disorder experienced a severe postnatal mental illness, including psychosis, mania, and/or hospitalization [17]. Often overlooked in the narrative of perinatal depression are the structural and social determinants such as poverty, education level, intimate partner violence, chronic stress from lower welfare support, poor social support, relationship factors, unemployment, neighborhood socio-economic status [18,19]. In addition, in certain regions such as South America and Africa, ethnicity has also been identified as a risk factor [18]. In South Asia and the Middle East, rigid cultural gender norms such as pressure to have a male child is also associated with PND [20,21].

Addressing the deeply rooted causes of maternal health inequities requires multiple and sustained interventions at every level.

## What has worked in the past?

The good news is that perinatal depression can sometimes be prevented through approaches that extend beyond clinical-based methods. However, much of the current evidence for preventing and treating perinatal depression comes from high-income countries. For example, the Reach Out, Stay Strong, Essentials (ROSE) program in the United States [22], designed for expectant mothers of newborns, offers prenatal sessions supplemented with a postnatal booster session. Topics covered include educating mothers about postpartum depression, guiding them through the challenges of transitioning into motherhood, managing relationships, self-care, and fostering assertiveness and goal-setting skills. Another program, Mothers and Babies (MB), employs cognitive-behavioral therapy principles in the United States to aid low-income, ethnically diverse perinatal women [23]. It has been culturally adapted and tested in various populations, including the Native American community. The intervention involves providing activities such as mindful art activities and story-telling to at-risk mothers throughout the perinatal period, aimed at improving maternal-infant bonding and preventing the onset of depression.

Community-based preventive perinatal depression programs, delivered by non-specialist health providers, offer an appealing approach that can be adapted and tested in LMICs. In low-income settings, there is a shortage of preventive perinatal depression programs, and relevant research remains limited. Although a few treatment-focused interventions in LMICs have been effective in treating perinatal depression using psychosocial therapies, such as the Thinking Healthy Programme (THP), which offers low-intensity psychological counseling by community health workers throughout the perinatal period. Initially tested in Pakistan and India, THP reduced the rate of perinatal depression by over half in the intervention group; at 12 months post partum, 73% of mothers who were assigned to the intervention group had recovered from their depressive disorder compared with 41% in the control group [24,25]. Nevertheless, preventive measures hold greater potential for primary healthcare integration compared to treatment interventions. This is partly due to such measures being more suitable for delivery within community settings. They also overcome the challenges of timely perinatal depression diagnosis that are particularly pronounced in low-income settings.

## Challenges and recommendations in mitigating perinatal depression in low-income settings

Despite the intent to address perinatal mental health, significant obstacles exist in combating perinatal depression in low-income settings:

1. **Dearth of research especially lived experiences of mothers**: The primary challenge of addressing perinatal depression in low-resource settings is the lack of research. To overcome this obstacle and facilitate effective interventions, it’s crucial to incorporate lived experiences when shaping pathways to care. Robust, context-specific, mother-centered research is essential to tailor interventions to the unique cultural and logistical realities of these regions, addressing both maternal and child well-being. Further, lived experience perspectives of emotional, physical, and social complexities of women could inform more targeted and empathetic care pathways in primary health settings.
2. **Provider Availability**: Another formidable challenge lies in ensuring that trained healthcare workers are available and have the resources to screen mothers and deliver psychosocial preventive and management interventions. It is critical to recognize the necessity of extending mental health support and education beyond specialists to ensure a wider reach. Equipping non-specialist health providers, including community health workers, with the knowledge and skills to offer such support is crucial to bridging that accessibility gap.
3. **Healthcare Sector Capacity**: The third challenge hinges on enhancing the capacity of the healthcare sector in low-income settings to integrate universal screening and support into routine maternal health programs. Following the World Health Organization-recommended stepped care model is a sensible approach. This model enables us to reach a broad spectrum of women with low-resource interventions while reserving specialist services for more complex cases. It’s a step towards resource-efficient and effective healthcare delivery. However, it’s evident that without written policies and dedicated national funding, a sense of urgency to implement such models remains conspicuously absent.

## Prioritizing perinatal mental health: A global imperative

The challenge of perinatal depression transcends borders; it’s a universal yet often hidden and complex concern that necessitates a collective, global response. As a global community, it is imperative that we address the issues surrounding perinatal depression by (a) raising awareness, (b) prioritizing funding for community-based mental health implementation and research, and (c) advocating for accessible mental health services. Stigma and deeply rooted biases conspire to keep women and their families silent, diverting the focus towards more visible health issues. However, we cannot allow this pattern to persist. We must take action to extend our support to the millions of women and children who suffer in silence each day, acknowledging that their struggles are no less urgent or deserving of attention than any other aspect of maternal and child health and well-being.

Together, we can ensure that no mother, regardless of her location, faces perinatal depression in isolation and that no child endures potential consequences of untreated perinatal depression. The mental health community can build a world where every mother and child receive the care and support, they so rightfully deserve.
